# *The Veterinary Quarterly* now ranked a top 10 journal in the Veterinary Science 2018 JCR

**DOI:** 10.1080/01652176.2019.1645453

**Published:** 2019-07-17

**Authors:** J. H. Han Van Der Kolk, Merran Govendir

**Affiliations:** a Swiss Institute for Equine Medicine (ISME), Vetsuisse Faculty, University of Bern, Bern, Switzerland;; b The Sydney School of Veterinary Science, Sydney, NSW, Australia

Dear Reader,

It is our pleasure to announce that *the Veterinary Quarterly* has seen its 2018 JCR Impact Factor increase from 1.492 to 2.340. As a consequence, it has also moved up in the rankings, and is now 9/141 in the Veterinary Sciences JCR Category (previously 41/140).

*The Veterinary Quarterly* is an international open access journal which publishes review articles and original research in the field of veterinary science and animal diseases.

The top cited article within the 2016 volume was by Zorriehzahra et al. (2016) entitled ‘Probiotics as beneficial microbes in aquaculture: an update on their multiple modes of action: a review’, whereas the top cited article within the 2017 volume was by Singh et al. (2017) entitled ‘Ebola virus - epidemiology, diagnosis, and control: threat to humans, lessons learnt, and preparedness plans - an update on its 40 year's journey’. Remarkably, both manuscripts were from the group led by Dr. Dhama from the Indian Veterinary Research Institute, Izatnagar, Bareilly in India. This effort by Dr. Dhama and his excellent team is greatly acknowledged and certainly contributed substantially to the increase in the JCR Impact Factor of *the Veterinary Quarterly* over the recent years.

In addition, the increase in *the Veterinary Quarterly* JCR Impact Factor also largely attributed to the great support from the authors, reviewers, and editorial board members. This is a good opportunity for Merran and myself to sincerely thank all *Veterinary Quarterly* reviewers and editorial board members for their thorough and timely comments that always improve the submitted manuscripts as well as the administration and editing teams at Taylor and Francis, the journal's publisher, also providing excellent support to the Editorial Board.

Last but not least, we welcome Drs. Martin Heinrichs, Muhammad Zubair Shabbir, and Vincenzo Tufarelli to our editorial board and wish them every success.

J. H. Han van der Kolk *Swiss Institute for Equine Medicine (ISME),
Vetsuisse Faculty, University of Bern, Bern,
Switzerland* johannes.vanderkolk@vetsuisse.unibe.ch Merran
Govendir *The Sydney School of Veterinary Science,* *Sydney, NSW,
Australia* merran.govendir@sydney.edu.au
